# Modelling the impact of vaccination on COVID-19 in African countries

**DOI:** 10.1371/journal.pcbi.1012456

**Published:** 2024-10-23

**Authors:** Dephney Mathebula, Abigail Amankwah, Kossi Amouzouvi, Kétévi Adiklè Assamagan, Somiealo Azote, Jesutofunmi Ayo Fajemisin, Jean Baptiste Fankam Fankame, Aluwani Guga, Moses Kamwela, Mulape Mutule Kanduza, Toivo Samuel Mabote, Francisco Fenias Macucule, Azwinndini Muronga, Ann Njeri, Michael Olusegun Oluwole, Cláudio Moisés Paulo

**Affiliations:** 1 Department of Decision Sciences, University of South Africa, Pretoria, South Africa; 2 Department of Mathematics, University of Cape Coast, Cape Coast, Ghana; 3 ScaDS.AI Dresden/Leipzig, TU Dresden, Dresden, Germany; 4 Department of Mathematics, KNUST, Kumasi, Ghana; 5 Brookhaven National Laboratory, Physics Department, Upton, New York, United States of America; 6 Department of Physics, Syracuse University, Syracuse, New York, United States of America; 7 Department of Physics, University of South Florida, Tampa, Florida, United States of America; 8 Molecular Sciences Institute, University of the Witwatersrand, Johannesburg, South Africa; 9 Department of Physics, University of Cape, Cape Town, South Africa; 10 Pharmacology Department, Lusaka Apex Medical University, Lusaka, Zambia; 11 Cancer Diseases Hospital, Lusaka, Zambia; 12 Department of Physics and Electronics, Rhodes University, Grahamstown, South Africa; 13 Department of Mathematical Sciences, University of South Africa, Florida, South Africa; 14 Faculty of Science, Nelson Mandela University, Gqeberha, South Africa; 15 School of Mathematics, Statistics and Physics, Newcastle University, Newcastle Upon Tyne, United Kingdom; 16 Department of Physics, University of Ibadan, Oyo, Nigeria; 17 Department of Physics, University Eduardo Mondlane, Maputo, Mozambique; Northeastern University, UNITED STATES OF AMERICA

## Abstract

The rapid development of vaccines to combat the spread of COVID-19, caused by the SARS-CoV-2 virus, is a great scientific achievement. Before the development of the COVID-19 vaccines, most studies capitalized on the available data that did not include pharmaceutical measures. Such studies focused on the impact of non-pharmaceutical measures such as social distancing, sanitation, use of face masks, and lockdowns to study the spread of COVID-19. In this study, we used the SIDARTHE-V model, an extension of the SIDARTHE model, which includes vaccination rollouts. We studied the impact of vaccination on the severity of the virus, specifically focusing on death rates, in African countries. The SIRDATHE-V model parameters were extracted by simultaneously fitting the COVID-19 cumulative data of deaths, recoveries, active cases, and full vaccinations reported by the governments of Ghana, Kenya, Mozambique, Nigeria, South Africa, Togo, and Zambia. Using South Africa as a case study, our analysis showed that the cumulative death rates declined drastically with the increased extent of vaccination drives. Whilst the infection rates sometimes increased with the arrival of new coronavirus variants, the death rates did not increase as they did before vaccination.

## Author summary

The onset of the COVID-19 pandemic prompted swift global responses. These included non-pharmaceutical control measures at the beginning of the pandemic such as social distancing, masks, and the later development of vaccines. In our first paper, we studied the evolution of the pandemic in the African continent by fitting the observed data with the SIRDATHE model in the first year (March 2020—March 2021) of the pandemic, before vaccination. We examined the impact of non-pharmaceutical control measures on COVID-19 transmission in the first year of the pandemic. As vaccination campaigns commenced, we extended our model to SIDARTHE-V model, which included the vaccination component. The SIRDATHE-V model assumes total immunity in the vaccinated population. However, our observations revealed breakthrough infections even amongst the vaccinated population. Therefore, in our application of the SIRDATHE-V model, we considered the fact that even the vaccinated could become infected.

In this study, we investigated the impact of COVID-19 vaccination in Africa. We analysed COVID-19 data from seven African countries: Ghana, Kenya, Mozambique, Nigeria, South Africa, Togo, and Zambia over approximately two years, from March 2020 to March 2022. Considering variations in vaccination programs in each country, for example, the use of different vaccines and different starting dates, this study focused on the impact of vaccination on the death rates.

Our findings showed a decline in cumulative death rates due to COVID-19 when vaccination drives were coupled with non-pharmaceutical control measures. We therefore conclude that in combating the spread of acute viral infections with dynamics similar to COVID-19, a comprehensive strategy involving both pharmaceutical (vaccination) and non-pharmaceutical (social measures) interventions is crucial.

## 1 Introduction

Since 2019, the severe acute respiratory syndrome coronavirus 2 (SARS-CoV-2) that causes the coronavirus disease 2019 (COVID-19) has been spreading worldwide [1]. To mitigate the spread of COVID-19, different control measures such as lockdowns, social distancing, face masks, sanitisers, and vaccination were implemented in countries across the globe [2]. Several COVID-19 vaccines, such as Pfizer and Johnson & Johnson, have been developed to combat the spread of COVID-19 [3]. These vaccines differ in their development, efficacy, storage, and administration [4].

While vaccination rollouts in developed countries commenced in mid-to-late 2020, vaccination campaigns in African countries began later in 2021 [5, 6]. Our study was informed by COVID-19 vaccination programs in seven African countries: Ghana, Kenya, Mozambique, Nigeria, South Africa, Togo, and Zambia. The commonly used vaccines in these countries were Pfizer, Johnson & Johnson, and Moderna. Because of the unequal availability of vaccines around the globe, different countries, including those in Africa, had different vaccine rollout dates which might impact a study such as this one [7, 8]. However, we analysed vaccination data for exactly one year in each of the seven countries, and therefore the starting dates of vaccination do not affect our conclusions.

The main objective of this study was to investigate the impact of COVID-19 vaccination in African countries using the SIDARTHE-V model [9]. This study is a continuation of our work reported in Ref. [10], Paper I hereafter. In Paper I, we studied the COVID-19 pandemic evolution in Africa Africa by collecting and analysing the COVID-19 data from nine African countries (Cameroon, Ghana, Kenya, Madagascar, Mozambique, Rwanda, South Africa, Togo, and Zambia) during the first year of the pandemic, March 2020—March 2021. The analysis was based on the SIDARTHE Model [11], which is one of the mathematical models used for studying disease outbreaks and pandemics. We estimated the time-dependent basic reproduction numbers, *R*_0_, and the fraction of infected but unaffected populations to offer insights into containment and vaccine strategies in African countries. We found that *R*_0_ ≤ 4 at the start of the pandemic but has since fallen to *R*_0_ ∼ 1. The unaffected fractions of the populations studied vary between 1 − 10% of the total recovered cases.

COVID-19 transmission rates across Africa varied over time because of changes in behaviours and government policies as the pandemic evolved, and the rollouts of COVID-19 vaccination programs in the second year of the pandemic. Subsequently, we modelled the outbreak and the impact of COVID-19 vaccination in seven African countries considering that several model parameters in the SIDARTHE-V model, varied over time. The seven African countries included in this study were purely selected based on COVID-19 data availability for a period of two years, March 2020—March 2022. ***Relevance of the study: A mathematical model was used to assess the spread of COVID-19 and the impact of COVID-19 vaccination in combating the spread of COVID-19 in African countries. This study is therefore crucial in assessing the impact of vaccination on transmission rates of other similar viruses and the health implications, such as government policies and pandemic preparedness in Africa***.

This paper is organized as follows: In Section 2.1, the formulation of the SIDARTHE-V model is presented, considering the impact of vaccination as reported in Ref. [9]. Section 3 provides the results and analysis of COVID-19 data with vaccination for the seven African countries considered in this study. Section 4 discusses the impact of vaccination on the spread of COVID-19 and death rates, and Section 5 presents the conclusions.

## 2 Methods

We investigated the impact of COVID-19 vaccination rollouts in Ghana, Kenya, Mozambique, Nigeria, South Africa, Togo, and Zambia. We analyzed COVID-19 data taken over two years, March 2020—March 2022. Our analysis considered two stages: without vaccination (first year of the pandemic, March 2020—March 2021); and with vaccination (the second year of the pandemic, March 2021—March 2022). We adopted and extended the SIDARTHE-V model [9] to extract and compare the model parameters to gauge the impact of vaccination as a pharmaceutical intervention.

### 2.1 SIDARTHE-V model with vaccination roll outs

**Fig 1:**
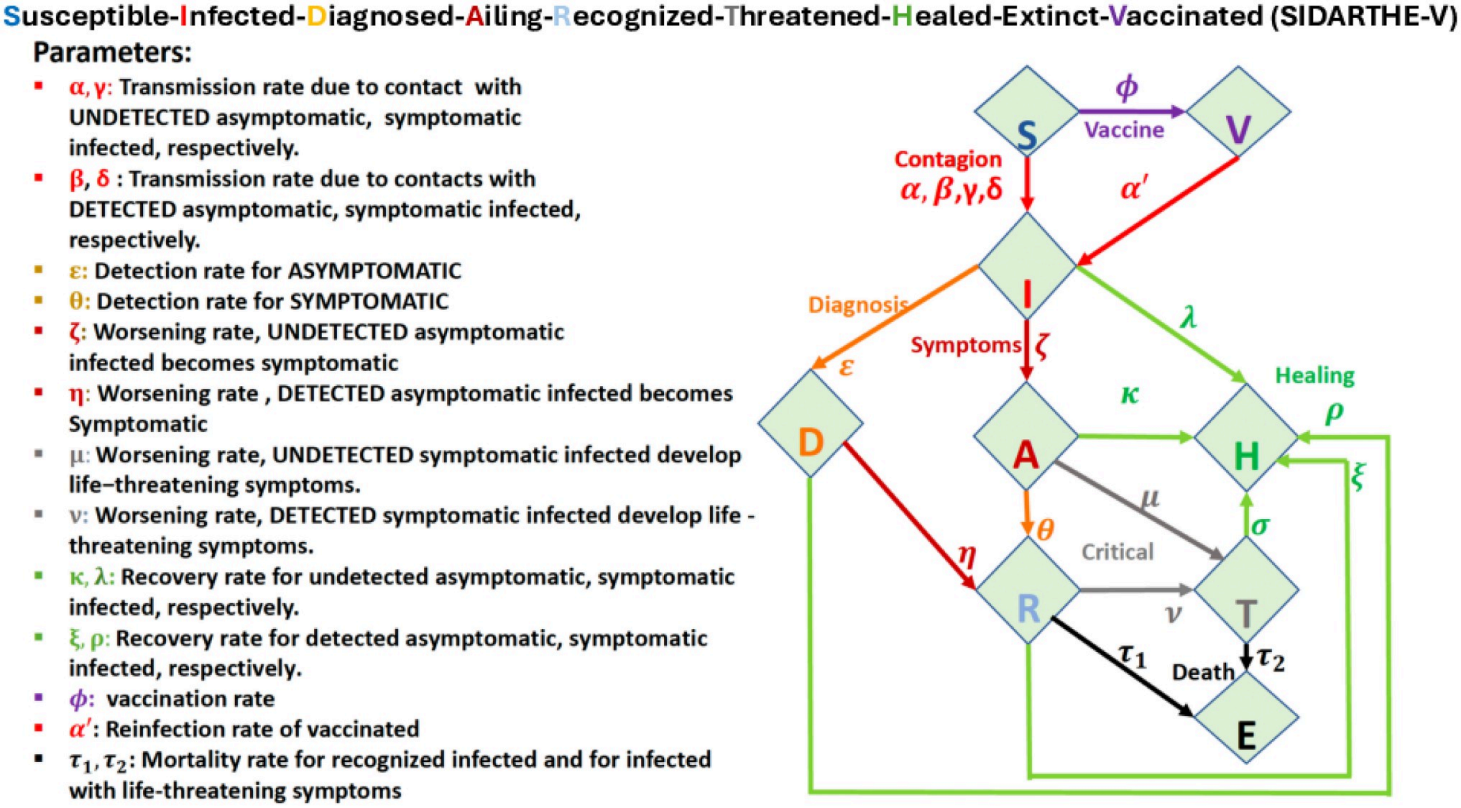
Flowchart illustrating the transmission dynamics of COVID-19 in the modified SIRDATHE-V model system, along with a description of the parameters used to fit the COVID-19 data. For further details, refer to Paper I Ref. [9].

In this study, we applied the SIDARTHE-V model [9], an extension of SIDARTHE model [11], with vaccination campaigns in the second year of the pandemic. Contrary to the original SIDARTHE-V, which assumes that all vaccinated individuals are immunized as reported in Ref. [9], in this study, we considered the possibility that vaccinated individuals can still get infected and become infectious. We captured these extra dynamics by connecting the vaccinated (V) and Infected (I) compartments, as shown in Fig 1. In Fig 1, we present the parameters and variables of the model as used in Eqs (1) and (2). Eq (1) describes the pandemic evolution, with vaccination rollouts, while Eq (2) describes the fraction of the population to be vaccinated to achieve herd immunity:

$$\begin{array}{c} \left\{ \begin{array}{ccc} \dot{S} & = & -(\alpha I+\beta D+\gamma A+\delta R)S-\phi S,\\ \dot{V} & = & -\alpha^{\prime }IV+\phi S,\\ \dot{I} & = & (\alpha I+\beta D+\gamma A+\delta R)S+\alpha^{\prime }IV-(\epsilon +\lambda +\zeta )I,\\ \dot{D} & = & \epsilon I-(\eta +\rho )D,\\ \dot{A} & = & \zeta I-(\theta +\mu +\kappa )A,\\ \dot{R} & = & \eta D+\theta A-(\tau_{1}+\nu )R,\\ \dot{T} & = & \mu A+\nu R-(\tau_{2}+\sigma )T,\\ \dot{H} & = & \lambda I+\kappa A+\sigma T+\xi R+\rho D,\\ \dot{E} & = & \tau_{1}R+\tau_{2}T, \end{array}\right. \end{array}$$ (1)

where the variables and the parameters are defined in Fig 1.

Our parameters are time-dependent, that is, the parameters are not constant and vary with time as the pandemic evolves. The time evolution of the pandemic is indicated by the dot over the variables in Eq (1). During learning, the model fine-tunes its parameters to optimize the fit to the data. Thus, the dynamics of the epidemic govern the collective temporal evolution of the parameters’ values. The parametric, basic reproduction number, *R*_0_, is the average number of secondary cases produced by an infected individual in a population where everyone is susceptible [12]. Estimating *R*_0_ helps in the implementation of appropriate responses to a pandemic evolution, in particular, the number of people to vaccinate for herd immunity. For the SIDARTHE-V model presented in Eq (1), *R*_0_ is given by:

$$\begin{array}{ccc} R_{0} & = & \frac{\alpha r_{2}r_{3}r_{4}+\beta \epsilon r_{3}r_{4}+\delta \epsilon \eta r_{3}+\delta r_{2}\tau \zeta +\gamma r_{2}r_{4}\zeta }{r_{1}r_{2}r_{3}r_{4}}, \end{array}$$ (2)

where *r*_1_ = *ϵ* + *ζ* + λ, *r*_2_ = *η* + *ρ*, *r*_3_ = *θ* + *μ* + *κ*, *r*_4_ = *ν* + *ξ*. Eq (2) shows that *R*_0_ is dependent on the model parameters that affect the evolution of a pandemic. Thus, it is very important to understand the model parameters and to ensure that the parameters are properly extracted. For a detailed description of the *R*_0_ derivation, we refer the reader to Ref. [11].

## 3 Results and analysis

In Paper I, we studied the impact of non-pharmaceutical control measures on the spread of COVID-19 in nine African countries. In this paper, we present the analysis of COVID-19 data from seven African countries, including the first year of the pandemic (without vaccination) and the second year of the pandemic (with vaccination). Nigeria’s COVID-19 data analysis was not included in Paper I. Therefore, we first present the analysis of the COVID-19 data from Nigeria for a period of two years, 2020-2022. This includes the first year without vaccination and the second year with vaccination.

### 3.1 COVID-19 data description

**Fig 2:**
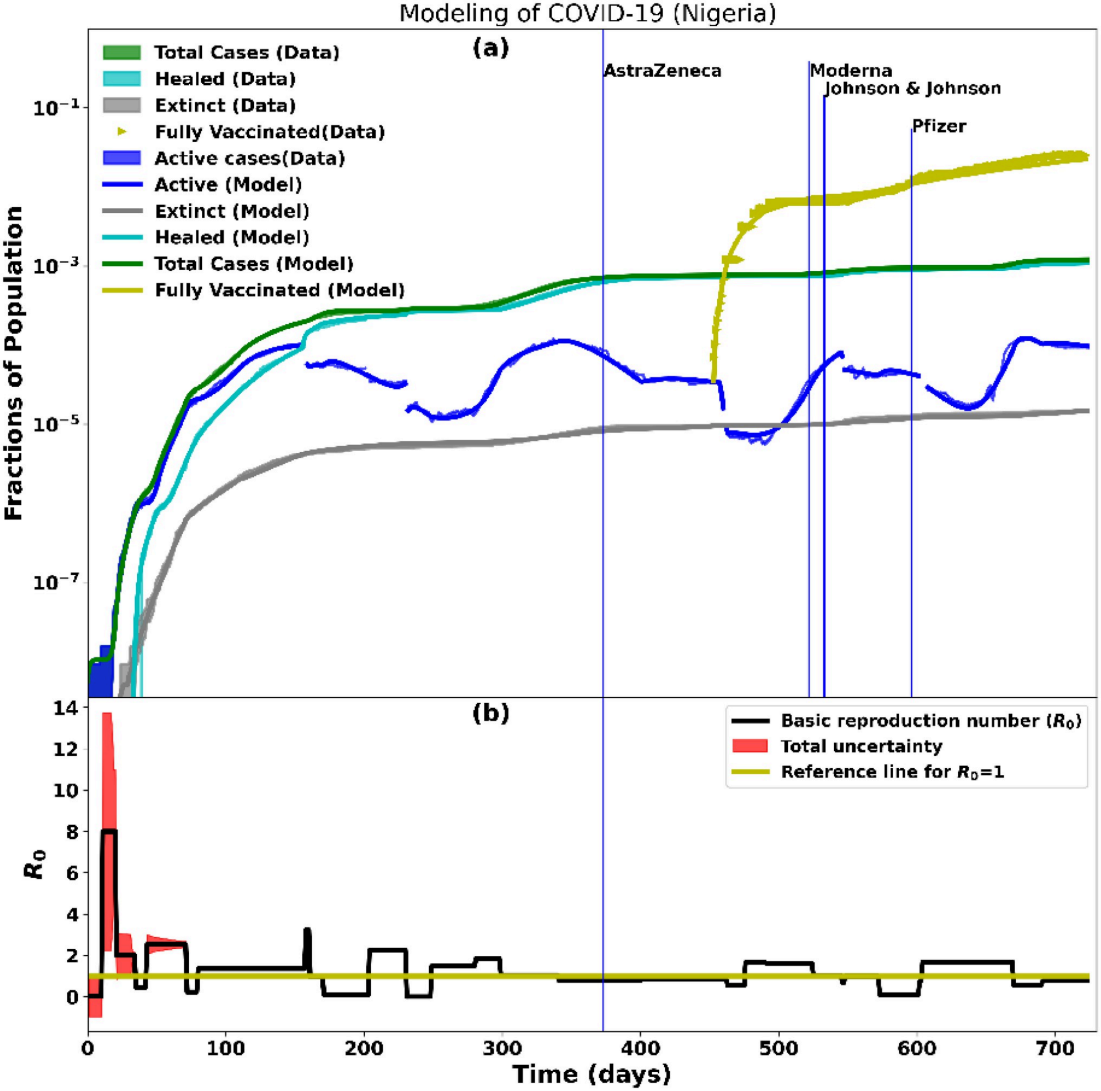
Modelling of ∼2 years of COVID-19 data for Nigeria. Day 0 corresponds to the onset of the pandemic on February 27, 2020. The top plot shows the data and the fitted SIRDATHE-V model for active, recovered, death, total cases, and fully-vaccinated individuals. The vaccination drive started on March 5, 2021. The bottom plot shows the time-dependent basic reproduction number, *R*_0_ which shows periodic minor rise and falls as the pandemic progressed, after the initial *R*_0_ = 8 at the onset of the pandemic.

**Fig 3:**
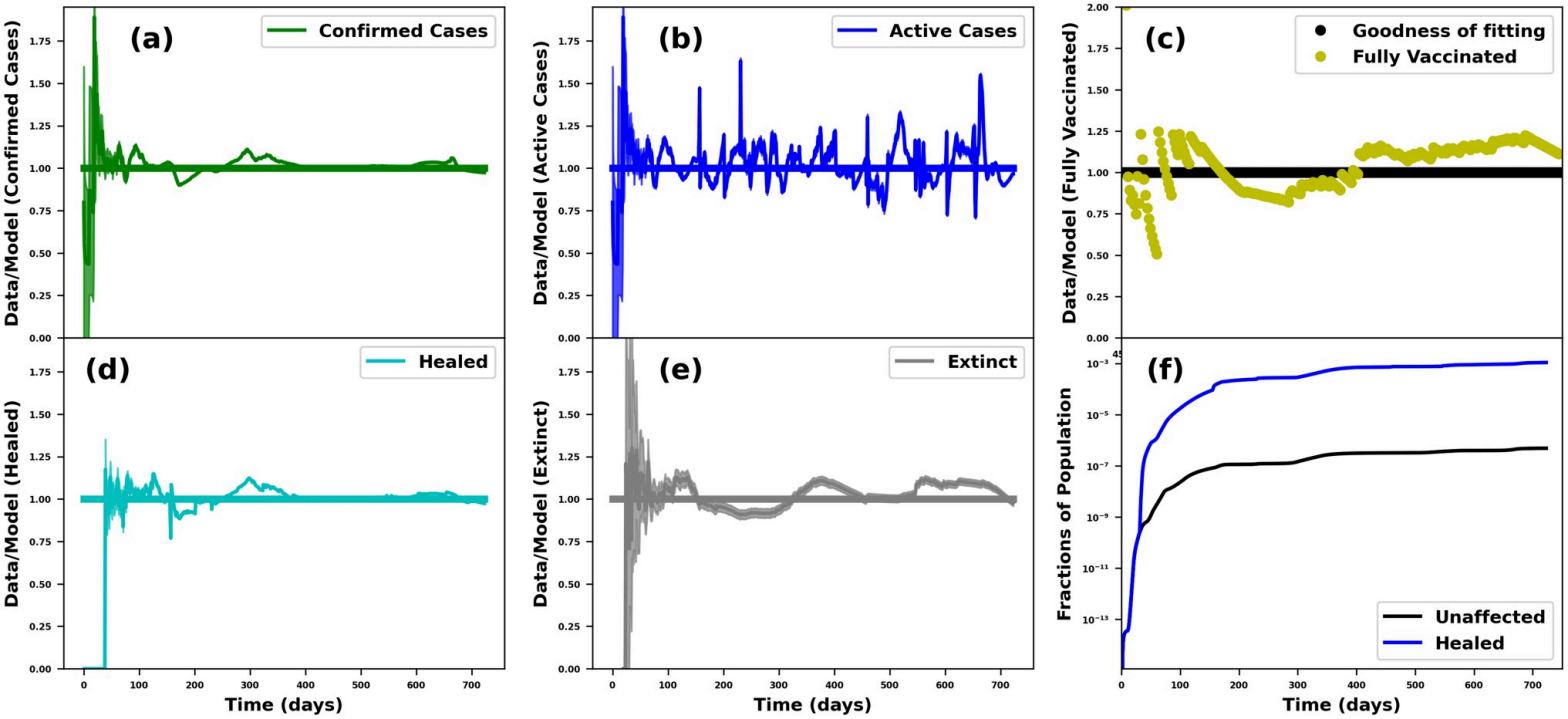
Goodness-of-fit on Nigerian data. The goodness-of-fit for the Nigerian data for confirmed (a), active (b), fully-vaccinated (c), healed (d), and dead/extinct (e) cases. The model prediction of the recovered population and the non-diagnosed fraction of the people that were infected and recovered without symptoms—this fraction, called the unaffected cases, is not measured nor included in the data, are shown in (f).

**Fig 4:**
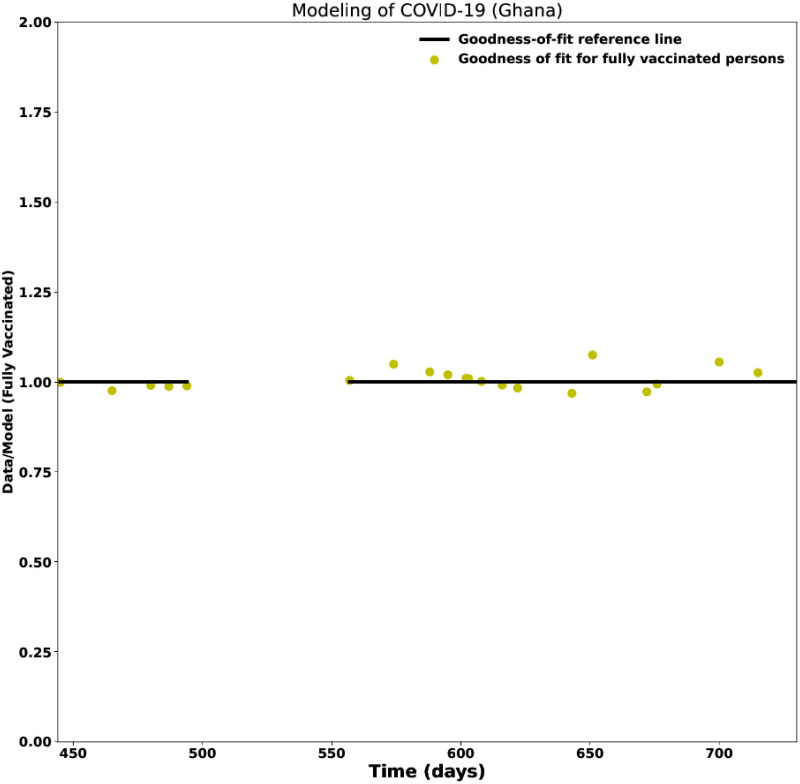
Goodness-of-fit on the fully-vaccinated data for Ghana. The goodness-of-fit on the fully-vaccinated data for from March 1, 2021, to February 28, 2022.

**Fig 5:**
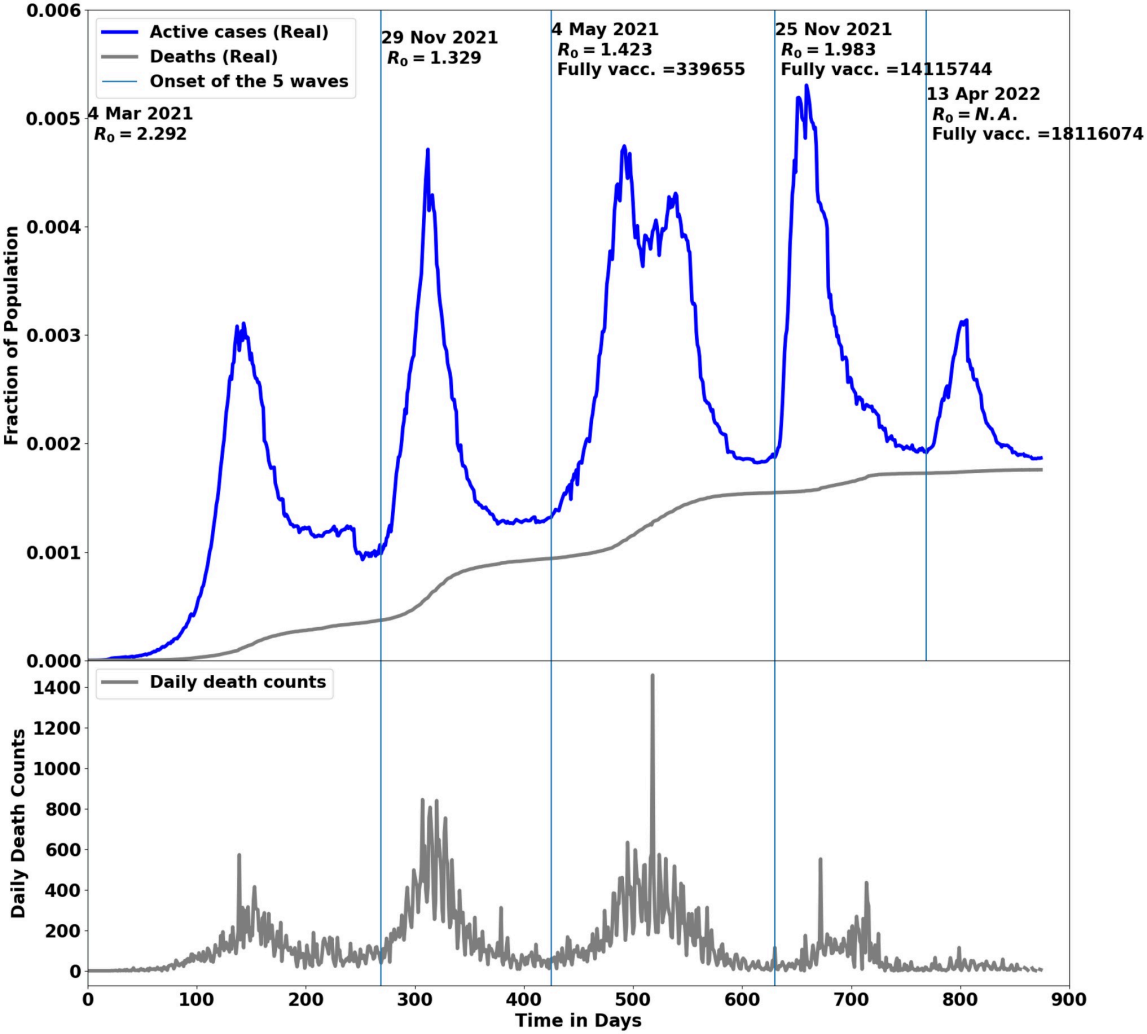
The COVID-19 waves in South Africa. The onset date for each wave and the *R*_0_ are also shown. At the onset of the third and fourth waves, the number of fully vaccinated individuals is also shown as these peaks were experienced during the vaccination period. The top plot shows the cumulative deaths as a function of time, while the bottom plot shows the daily death counts for a period of two years, March 2020—March 2022. The vertical lines indicate the beginning of each pandemic wave. The data for the other African countries is presented in Table 1.

The observed COVID-19 data (total confirmed, active, healed, extinct, and vaccinated) from each of the seven countries was recorded and analysed. These data were then fitted using the SIRDATHE-V model as described in Section 2.1. The observed data and the fitted parameters were plotted for each country as a function of time (from Day 0 of the pandemic). We present these data in Figs 2–5. The relative statistical uncertainties are shown by the error bars on the data. Indeed, by assuming that there was no systematic uncertainty induced by the model and that the observed (total confirmed, active, healed, extinct and vaccinated) data followed a Poisson distribution, the uncertainty is defined by $\Delta D=\frac{\sqrt{D}}{N}$ where *D* is the accumulated observed (total confirmed, active, healed, extinct, or vaccinated) data and *N* is the total population. Thus, the error bar is given by *D* ± Δ*D*. Consequently, the error bars vanish when Δ*D* approaches zero.

To assess the accuracy of the fit of the model to the data, we derive the goodness of fit between the COVID-19 data and the model. We present the goodness-of-fit for the Nigerian (see Fig 3) and Ghanaian (see Fig 4) data as examples. The goodness of fit was given by the ratio of the observed data to the fitted model. A good fit was expected to align the time-dependent ratios to 1 when the data and the model agree.

To fully comprehend the situation in South Africa, we analysed the observed new COVID-19 waves as a result of new and emerging coronavirus variants. Fig 5 highlights the onset dates for each wave, the value of *R*_0_, at each onset, and the total number of fully vaccinated at the onset of each new wave. For the other 6 African countries, we present the data for the 3^*rd*^ and the 4^*th*^ waves in Table 1: the wave onset date, *R*_0_, fraction of the population that must be vaccinated, the fraction of the population that was fully vaccinated and lastly, whether herd immunity was reached in each country. Contrary to focusing on the original dynamics of the disease as depicted in Fig 1, starting from February 18, 2021, we assumed that; i) the coronavirus mutations had stopped, ii) all the pharmaceutical and non-pharmaceutical interventions remained the same, and, iv) the host population was quarantined.

**Table 1:** Countrywise vaccination status at the onset of the 3rd and 4th waves.

Country/Population	Waves	Onset date	R_0_	Must be vacc.	Fully vacc.	Herd Imm.?
Ghana	3^rd^ Wave	03.06.2021	1.24	5.77M	0.376M	False
29.8M	4^th^ Wave	30.11.2021	1.03	0.75M	0.842M	True
Kenya	3^rd^ Wave	03.07.2021	1.50	15.70M	0.52M	False
47.0M	4^th^ Wave	25.10.2021	1.18	7.04M	1.78M	False
Nigeria	3^rd^ Wave	30.06.2021	1.66	85.89M	1.16M	False
217M	4^th^ Wave	21.10.2021	1.67	87.24M	3.52M	False
Mozambique	3^rd^ Wave	27.05.2021	2.33	16.55M	0	False
29M	4^th^ Wave	14.11.2021	2.68	18.17M	2.7M	False
Zambia	3^rd^ Wave	21.05.2021	1.09	1.38M	0.001M	False
17.5M	4^th^ Wave	09.12.2021	1.80	7.78M	0.73M	False
Togo	3^rd^ Wave	06.04.2021	4.45	6.20M	0.04M	False
8.0M	4^th^ Wave	07.11.2021	0.91	0.83M	0.47M	False

‘Must be vaccinated’ (abbreviated as ‘Must be vacc.’) is the number of the population to be vaccinated to reach herd immunity (abbreviated as ‘herd Imm.’). Herd Imm. is reached when the ‘Must be vaccinated’ is less or equal to fully vaccinated (abbreviated as ‘Fully vacc.’), or if *R*_0_ ≤ 1.

#### 3.1.1 COVID-19 vaccination analysis for Nigeria

In Nigeria, the first COVID-19 case was reported on February 27, 2020 in Ogun State. An airplane from Milan, Italy, arrived at the Murtala Muhammed International Airport, Lagos, on February 14, 2020, with an infected Italian citizen. The health authorities (Nigeria Centre for Disease Control) implemented containment measures by contact tracing of ‘Persons of Interest’ which included all persons on this particular flight and those in close contact with the first patient [13]. After two weeks, other cases were detected in Lagos and Abuja.

In response, the Federal Government restricted international commercial flights into the country, effective from March 23, 2020 [14]. The Government also ordered the closure of schools and all non-essential services (businesses and industries) and ordered the cessation of all movements in Lagos State, Ogun State, and the Federal Capital Territory, Abuja, on March 29, 2020, for an initial period of 14 days. Later, the restrictions on movement were extended for another 14 days from April 12, 2020 [15]. Most State Governments restricted public gatherings and religious activities for over 50 people. The Federal Government banned domestic flights on April 20, 2020, and ordered a nationwide overnight curfew from 8:00 pm to 6:00 am on May 2, 2020. On May 6, 2020, the Government announced an extension of the travel ban on both international and local flights to, June 7, 2020. International flights resumed on August 29, 2020 [16]. On September 3, 2020, the overnight curfews were later eased to 12:00—4:00 am. On September 6, 2020, the Federal Government authorized the gradual easing of lockdown in the previously restricted states and mandated the use of face masks in public.

On January 27, 2021, the President signed six COVID-19 Health Protection Regulations 2021, with restrictions on gatherings, operations of public places, mandatory compliance with treatment protocols, offenses, and penalties, enforcement, and application, and lastly, the interpretation and citations of the regulations [17].

As of March 27, 2022, about two years since the first COVID-19 case was reported in the country, the total number of confirmed cases was 255,341. A total of 249,566 cases had been discharged from the hospital, with current active cases at 2,633 and total deaths at 3,142. The first COVID-19 related death in the country was reported on March 23, 2020. COVID-19 testing began on April 8, 2020, and by March 27, 2022, 4,589,725 tests had been carried out [14, 15].

The first shipment of four million Oxford-AstraZeneca COVID-19 vaccines [18] arrived in the country on March 2, 2021, and vaccination began on March 5, 2021. The country received subsequent shipments of Moderna, Johnson & Johnson, and Pfizer COVID-19 vaccines [18] on August 1, August 12, and October 14, 2021, respectively. Due to the single dose requirement of the Johnson & Johnson COVID-19 vaccine, Nigeria’s National Primary Health Care Development Agency (NPHCDA) prioritised hard-to-reach and vulnerable areas for vaccination [19]. As of March 27, 2022, 21,049,754 persons had received their first dose, and 9,565,143 had received a second dose [19]. As the number of vaccinated individuals increased, the overall infection rates decreased. This allowed the government to gradually ease the strict lockdown measures and restrictions on businesses, public gatherings, and travel [20].

Fig 2 (top plot) shows the SIDARTHE-V modelling of the Nigerian COVID-19 data of active, recovered, extinct (death), and fully-vaccinated cases, while the bottom plot shows the time-dependent basic reproduction number, *R*_0_. *R*_0_ was obtained by fitting the model to the data. *R*_0_ increased steadily to ∼8 after the first week of the onset of the pandemic. This can be attributed to the initial phase of the pandemic and the lack of effective public control measures. Around day 35, *R*_0_ < 1 was due to the introduction of public control measures by the government and awareness by the public. Another increase in *R*_0_ > 2 was observed around day 40 most likely due to the difficulties in complying with the control measures. *R*_0_ > 3 at day 150 due to the ineffectiveness of the measures in some parts of the country and the lack of enforcement strategies from the government. Around day 165, *R*_0_ dropped below <1 and increased above >2 around day 205. Another drop occurred around day 230 to *R*_0_ = 0 after more restrictions were imposed by the government. We see that around day 250, there was an increase in *R*_0_ > 1. At day 700, *R*_0_ < 0. These fluctuations in *R*_0_ could be attributed to easing in restrictions and a lack of adherence to COVID-19 control measures. The goodness-of-fit of the model on the COVID-19 data is poor at the onset of the pandemic but our fitting improves with time as we obtained more data as shown in Fig 3.

#### 3.1.2 Analysis of COVID-19 data with vaccination for Kenya

The data used in this analysis were taken from the daily press releases by the Ministry of Health, Government of the Republic of Kenya [21]. Having received the first 1.12 million doses of the Oxford-AstraZeneca vaccine, the vaccination drive in Kenya kicked off on March 5, 2021. This was one year after the first COVID-19 case was reported on March 12, 2020. Six hundred and sixty-seven doses of AstraZeneca were administered on the first day of vaccination to front-line healthcare workers only at the Kenyatta National Hospital in Nairobi. This was then followed by other essential workers such as security officers and teachers in the first few weeks of the vaccination program, followed by targeted people with higher risks of severe disease and those aged 50 years and above. The second dose was administered on May 28, 2021, and 203 people received their second dose.

After five months of administering the AstraZeneca vaccine, 880,460 doses of the Moderna vaccine were received on August 23, 2021, from the US government via COVAX, making Moderna the second COVID-19 vaccine to be offered in the country. Additional 141,600 doses of Johnson & Johnson vaccine were received soon afterward on September 3, 2021. This was the third vaccine type to be offered and totaled 4.2 million doses of vaccine received [21]. On September 17, 2021, the country received 795,600 doses of the Pfizer vaccine from the US government, making Pfizer the fourth vaccine offered. Shortly afterward, on September 18, 2021, 200,000 doses of the Sinopharm COVID-19 vaccine were received from the Chinese government. The government had authorised all five vaccines and at the time of writing, they were being used across the country.

**Fig 6:**
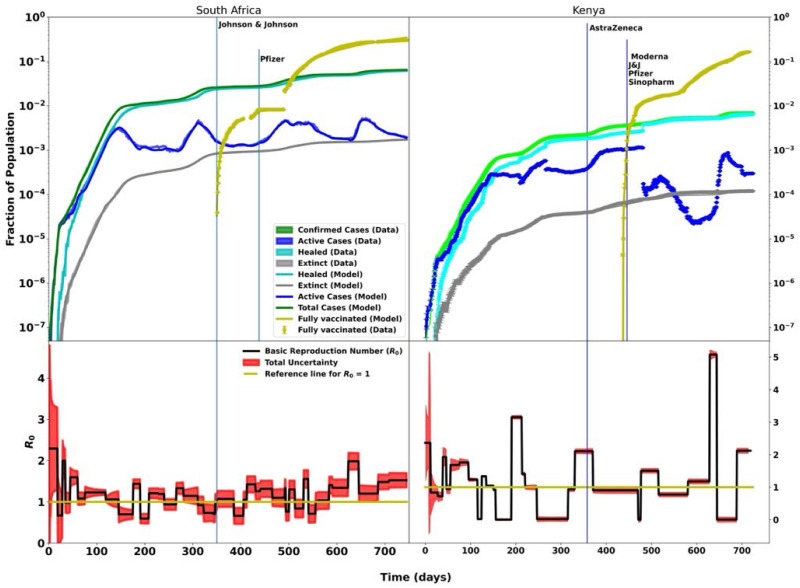
The modelling of ∼2 years of COVID-19 data for South Africa (left panel) and Kenya (right panel). Day 0 corresponds to the onset of the pandemic, that is, March 5 and March 12, 2020, for South Africa and Kenya, respectively. The top plots show the data and the model fitting for the active, recovered, death, total cases, and fully-vaccinated individuals. Vaccination drives started on February 28, 2021, in South Africa and March 5, 2021 in Kenya. The bottom plots show the time-dependent basic reproduction numbers, *R*_0_, which varied over time as the pandemic evolved.

After a slow uptake of the vaccines among the population due to vaccine hesitancy [22], a spike was observed on November 23, 2021, with the highest number of vaccination doses administered to 103,506 people in a single day. This followed a government directive on November 21, 2021, stating that anyone not vaccinated by December 21, 2021, would be refused in-person government services and access to public entertainment spots such as restaurants. As of December 2021, 7% of the population was fully vaccinated and ∼10% of the population was partly vaccinated. This figure slightly surpassed the government target of 10 million people by the end of the year. Fig 6 (right panel) shows the modelling of two years of COVID-19 data in Kenya with the vaccination rolls commencing on day 358 (highlighted by the blue vertical line), almost a year after the first COVID-19 case was reported—a detailed study of the data before vaccination campaigns were discussed in Paper I. The issuance of the second dose began around day 450 as highlighted by the second blue vertical line. Around day 480 (∼30 days after the second dose), there was a sharp decrease in active cases. Into the second year of the COVID-19 pandemic, the basic reproduction number *R*_0_ remained ≈1 or below 1 with slight variations during minor peaks. At day ∼ 650, *R*_0_ increased sharply to ∼5. This was due to a slight but sharp increase in active cases, following a steady decrease in active cases in the country.

Kenya is part of the WHO AFRO 20 priority African countries with the slowest rates of COVID-19 vaccination uptake [23]. Therefore, the WHO AFRO implemented phased COVID-19 vaccination campaigns in February 2022 to boost vaccination rates. This entailed community outreach efforts and increased vaccination sites from 800 to 6,000 sites. Over two weeks (February 3–17), the daily vaccination average increased from 70,000 to 200,000 people. This also raised the percentage of the population that was fully vaccinated from 9.9% to 13.4%. As of March 11, 2022, two years after the first COVID-19 case was reported and one year after the mass vaccination program rollout, 8,054,405 vaccine doses were administered and ∼14.8% (7,930,000) of the population was fully vaccinated. At the time of writing, a total of 323,140 COVID-19 cases had been reported, and a total of 5,644 deaths recorded.

COVID-19 restrictions are no longer in place although the government is encouraging citizens to wear masks and maintain social distancing where possible. Factors affecting the vaccination program in Kenya included: i) funding, ii) the availability of vaccines, iii) storage requirements, iv) vaccine hesitancy amongst the population [22] and geographical inequalities in accessing vaccines in hard-to-reach areas [24]. The government aimed to vaccinate 15.91 million people by June 2023 in a 3-phased rollout approach initially targeting 1.25 million people by June 2021 in phase one. This was followed by phase two, July 2021–June 2022, with a target of 9.76 million people, including the elderly and people with underlying health conditions. The third phase started in July 2022 and will run until June 2023, with a target of 4.9 million people above 18 years old, those with underlying health risks, and essential workers.

#### 3.1.3 Analysis of COVID-19 data with vaccination for Ghana

**Fig 7:**
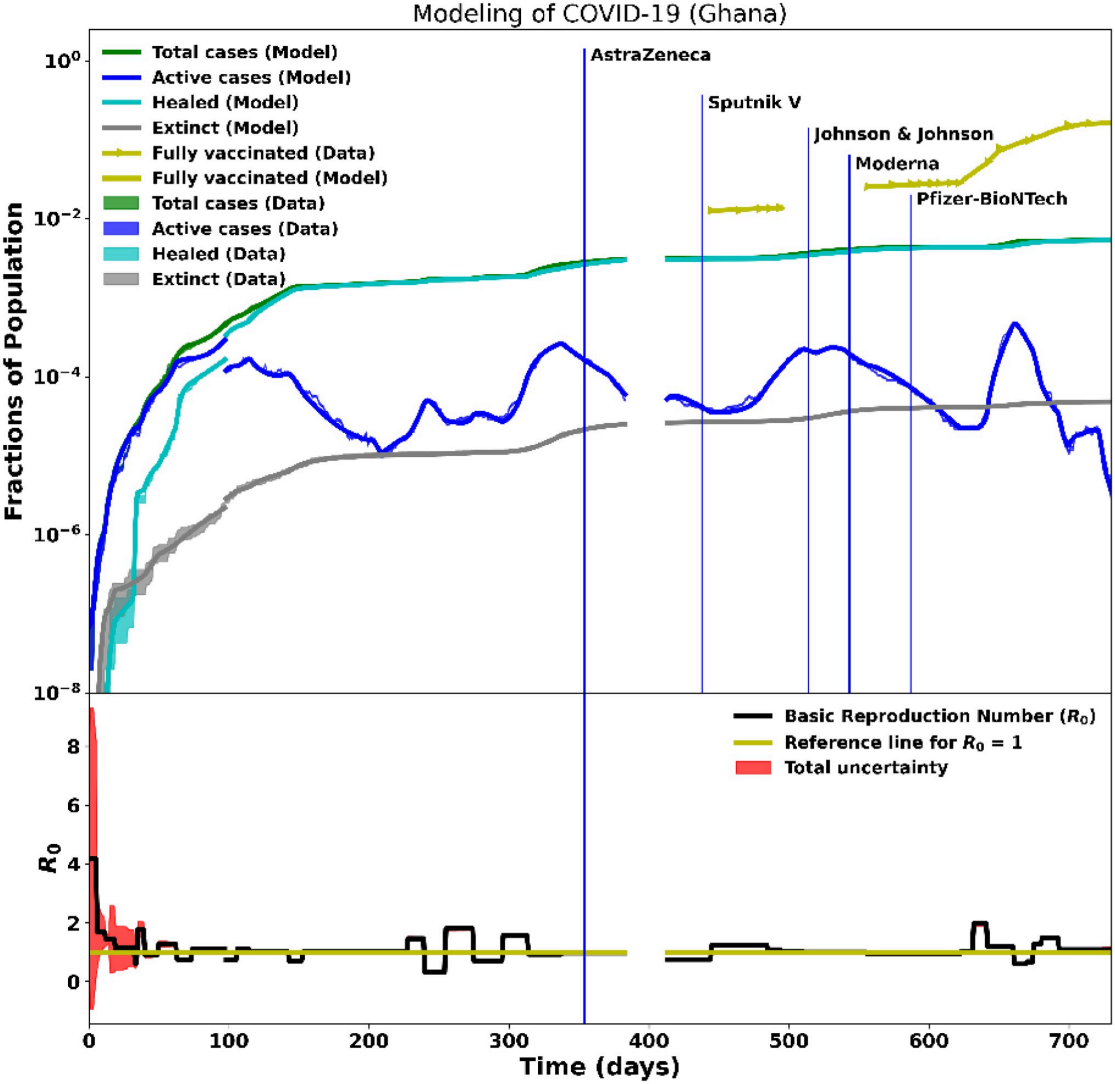
Modelling of ∼2 years of COVID-19 data for Ghana. Day 0 corresponds to the onset of the pandemic on March 12, 2020. The top plot shows the data and the fitted model for the active, recovered, death, total cases, and fully-vaccinated cases. Vaccination drives started on March 1, 2021. The bottom plot shows the time-dependent basic reproduction number, *R*_0_, and how it varied with time as the pandemic evolved. At the pandemic onset, *R*_0_ > 4 as no containment measures had been put in place and gradually dropped to zero in the same instances, showing the effectiveness of the containment measures implemented.

In Ghana, the government committed to acquiring COVID-19 vaccines on December 20, 2020 [25]. The arrival of the first shipment of COVID-19 vaccines, from the COVAX initiative to African countries, enabled Ghana Health Authority to begin its first vaccine rollout on March 1, 2021, with the AstraZeneca vaccine [26–28]. The president and the vice president were the first to receive the AstraZeneca vaccine on March 1, 2021 [29]. In addition, Johnson & Johnson (J&J), Moderna, Pfizer, and Sputnik V vaccines were also approved and widely administered. Fig 7 shows the modelling of the Ghanaian data over two years: data from the first year of the pandemic—before vaccination, were analysed, and discussed in Paper I. In this study, we focused on the second year of data with vaccination. Fig 4 shows the fitness of good of the model on the data for the fully-vaccinated persons during the vaccination campaigns.

Four COVID-19 waves were recorded in Ghana. These waves were caused by the emergence of Beta, Alpha, Delta, and Omicron coronavirus variants. These four waves are characterised by the four peaks in Fig 7 (top plot). A study conducted in Ref. [30] indicated that Delta and Alpha were among the most viral variants in Ghana. At the time of writing, the Beta variant was still being monitored in Ghana since it had the third-highest frequency. During the second wave, regions further from Accra, such as the Northern and Upper East, had different variants. These locations lagged behind the rest of the country in the third wave and did not appear to experience any waves [31]. The Beta variant was prominent after international flights resumed in September 2020, with the related reproduction number *R*_0_ decreasing from August 2020 as shown in Fig 7 (bottom plot). The Alpha variant superseded the Beta variant in January 2021 and became the major cause of all reported illnesses until June 2021, when Delta lineages took over until December 2021. The major coronavirus variants were first detected in travellers before being reported in communities [30].

By April 25, 2022, 14,268,269 doses of vaccines had been administered, with 18.3% of the population being fully vaccinated and 29.9% having received at least one dose of a vaccine. Furthermore, 360,201 people had received the first booster dose. By April 30, 2022, there were 161,216 confirmed cases in Ghana. Out of these, there were 159,737 recoveries, 1,445 deaths, and 34 active cases. Great Accra region alone reported 56.3% of the total cases due to the high population density. This was followed by the Ashanti region with 13.8% of the total cases [31].

#### 3.1.4 Analysis of COVID-19 data with vaccination for Mozambique

**Fig 8:**
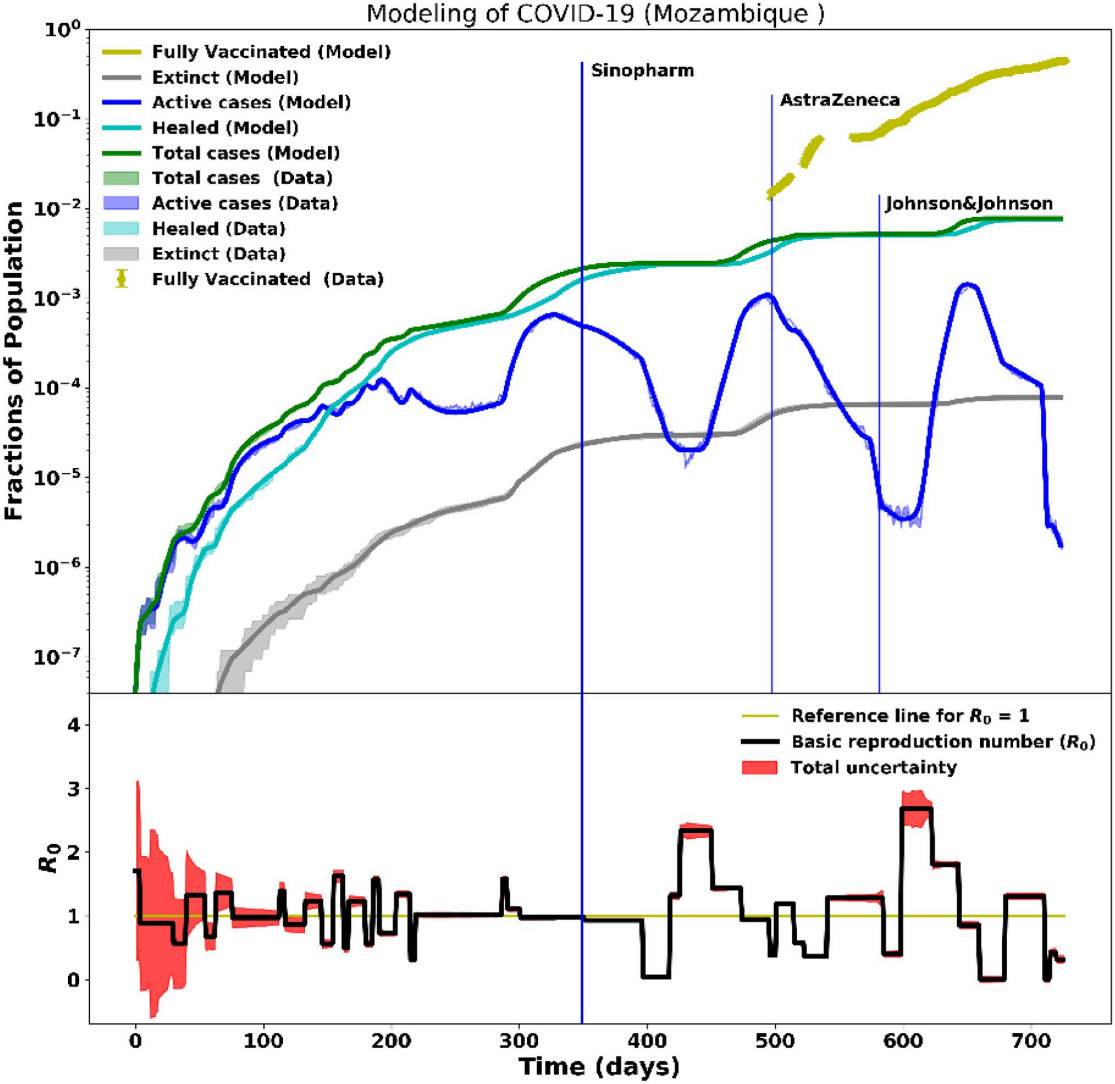
Modelling of ∼2 years of COVID-19 data for Mozambique. The modelling of the COVID-19 data took into account the vaccination campaign in Mozambique. Day 0 corresponds to the onset of the pandemic on March 20, 2020. Following similar procedures from the previous works done in our Paper I, the top plot presents the data and model for some of the main pandemic parameters analyzed (that is, active, recovered, death, and total cases) and now includes the fully-vaccinated data. The vaccination drives started on March 8, 2021. The bottom plot shows the time-dependent basic reproduction number.

The datasets used in this study for the particular case of Mozambique were obtained from the daily press releases and daily bulletins on the website of the government [32, 33]. Modelling of COVID-19 data was carried out in this work with the main purpose of understanding the vaccination impact during the pandemic evolution in the country, that is, in the second year of the pandemic. Fig 8 shows the fitting of the COVID-19 data for two years; first year without vaccination (analysed and presented in Paper I) and results for approximately one year with the vaccination drives.

In Mozambique, the vaccination campaign started on March 8, 2021. During this period, there was already a reduction of active cases because of non-pharmaceutical measures that were being implemented according to Decree 7/2021 of March 5 (see Ref. [34]). In general, the first vaccination campaign targeted health professionals, older people, diabetic patients, defense and security forces, as well as university staff [35]. Between April 19 and May 10, 2021, Mozambique had the second stage of vaccination that covered final-year medical students, university staff not covered during the first stage, inmates, police, and primary school teachers. The third stage of vaccination was between October 20 and November 3, 2021; it covered carers, people who were not vaccinated in the first two stages, motorcycle taxis, students, and all vulnerable people. Around the end of the fourth wave, on January 23, 2022, booster doses were introduced [36].

Fig 8 (bottom plot) shows the time-dependent *R*_0_ in Mozambique. The *R*_0_ varied from 2.5 to 0.1 as follows: 1) during the second wave, the *R*_0_ varied between 0.1 to 2.1; 2) in the third wave, the *R*_0_ varied between 0.4 to 2.5; 3) in the fourth wave, the *R*_0_ varied between 0.1 to 1.8. The fluctuations in *R*_0_ were related to the Government regulations of non-pharmaceutical interventions together with the onset of new variants which triggered new waves.

**Fig 9:**
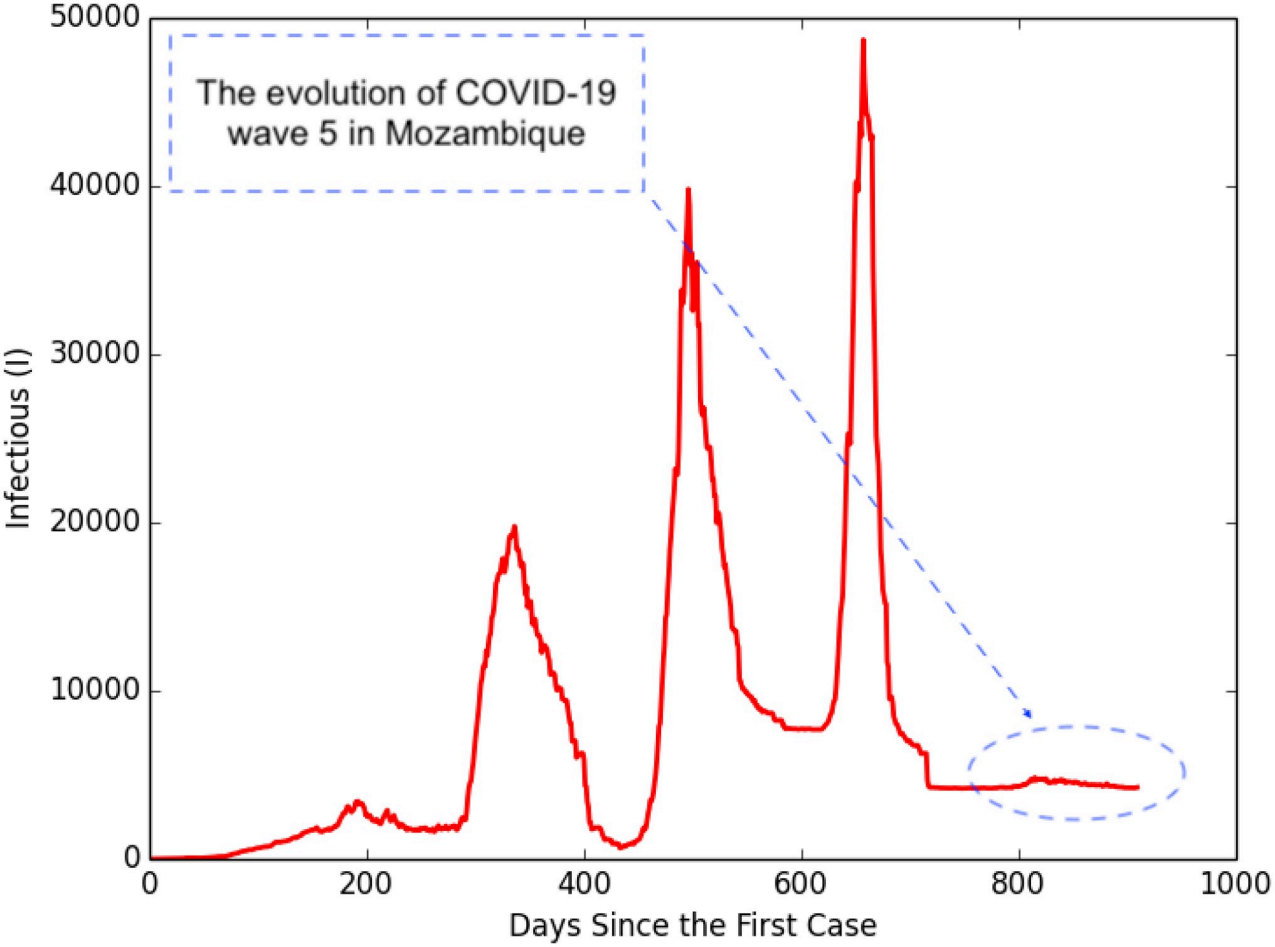
The COVID-19 pandemic waves observed in Mozambique. The fifth wave occurred during the vaccination campaigns and was relatively smaller compared to the previous three waves.

During the vaccination campaigns, new infections were still emerging, but with diminishing impact as shown in Fig 9—the fifth wave of the pandemic started in the last week of May 2022 and was fading in the time of writing. The onset of this wave, relatively small (duration and impact) compared to the previous ones, coincided with a time when the winter season brought unusually low temperatures in some regions and many people suffered from typical flu symptoms. The death rate in this wave was also very low, whilst the recovery rate was high, with a few people requiring hospitalization.

The Mozambican government had set a vaccination target of 15 million people at the time of writing. In this period, about 97% were fully vaccinated—of these, 614,842 individuals had received a booster and the government was planning to start vaccinating people aged 12 to 17 years [32, 33].

#### 3.1.5 Analysis of COVID-19 data with vaccination for Togo

On March 7, 2021, approximately one year after the first case was reported in Togo, the country received 156,000 doses of AstraZeneca through the COVAX facility [37, 38]. The vaccination campaign commenced immediately on March 10, 2021. An additional 120,000 doses of AstraZeneca were received on March 31, 2021. Afterwards, 100,620 Pfizer doses were obtained in April 2021, followed by 200,000 doses of Sinovac on May 23, 2021. On August 7, 2021, the country received an additional 118,000 doses of the Johnson & Johnson vaccine out of the 4 million doses that had been ordered. As of August 14, 2022, the World Health Organisation Coronavirus Dashboard indicated that Togo had received 3,262,548 COVID-19 vaccine doses, with 2,152,846 people vaccinated–corresponding to ∼25.4% of the population qualified for vaccination–with 1,425,113 persons fully vaccinated [39].

**Fig 10:**
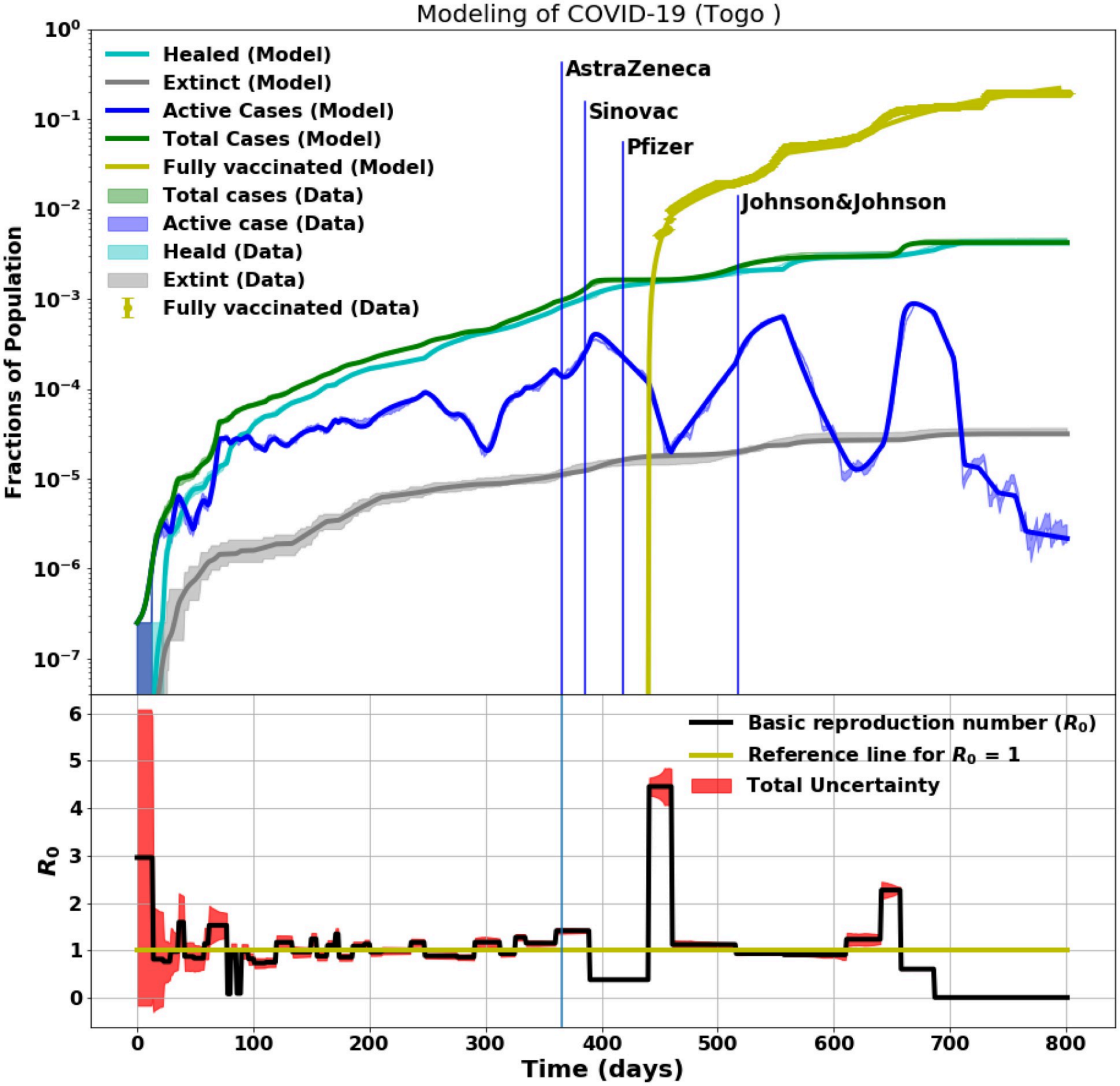
Modelling of ∼2 years of COVID-19 data for Togo. Day 0 corresponds to the onset of the pandemic on March 6. The top plots show the data and the fitted model for the active, recovered, death, total cases, and fully-vaccinated cases. Vaccination drives started on March 9, 2021. The bottom plot shows the time-dependent basic reproduction number, *R*_0_, and how it varied with time as the pandemic evolved starting at *R*_0_ ∼ 4 in Togo at the pandemic onset.

We modelled the COVID-19 data from Togo as shown in Fig 10 (top plot). The data from the first year of the pandemic–before vaccination–were analysed and presented in Paper I. The vaccination campaigns started with the health workers on March 10, 2021, day 370 of the pandemic, as shown in Fig 10. This was then followed by clinically vulnerable individuals and citizens over the age of 50 years [37, 38]. By May 2021, 93 percent of the healthcare workers had received their second dose [40]. One month after the vaccination campaign (from day 400) began, the impact on the infection rate is reflected in *R*_0_ (see Fig 10, bottom plot). Active cases continued to decrease up to three months after the vaccination drives commenced, while *R*_0_ sharply increased in the third month (on Day ∼450). This increase in *R*_0_ resulted from the relaxation of the control measures that were in place before the start of the vaccination. These control measures were largely ignored, as people assumed that the problem of COVID-19 would be solved immediately by the arrival of the vaccines. After day 470, the active cases started to increase again when the vaccine doses were finished and a new COVID-19 variant (Delta) emerged. As the active cases began to rise, the government warned the population of the new variant and encouraged rigorous adherence to the control measures. More vaccines were received and distributed across the country.

However, as the government accelerated the vaccination campaign, vaccine hesitancy set in [41–43]. Measures to encourage vaccination were therefore put in place, such as obligatory presentation of the COVID-19 vaccination card before entering any public institution. Despite these different strategies, as of September 17, 2021, the fraction of the population that had received two doses of the COVID-19 vaccine was only 5.6%. To reach the vaccination target, the WHO Country Office in Togo provided technical and financial support to the Togolese government. Through the Ministry of Health, Public Hygiene, and Universal Access to Health Care, they initiated community dialogues and broad awareness-raising in the Grand-Lomé region, the epicenter of the epidemic. This reduced misinformation and removed barriers to vaccine acceptance. However, there have been periodic rises and falls in the basic reproduction number, as seen in the bottom plot of Fig 10. The peaks may be related to ignoring the COVID-19 control measures such as social distancing by the public. This overall observation allows us to stress that both control measures and vaccination are necessary to overcome the COVID-19 pandemic and any other similar pandemics.

#### 3.1.6 Analysis of COVID-19 data with vaccination for Zambia

**Fig 11:**
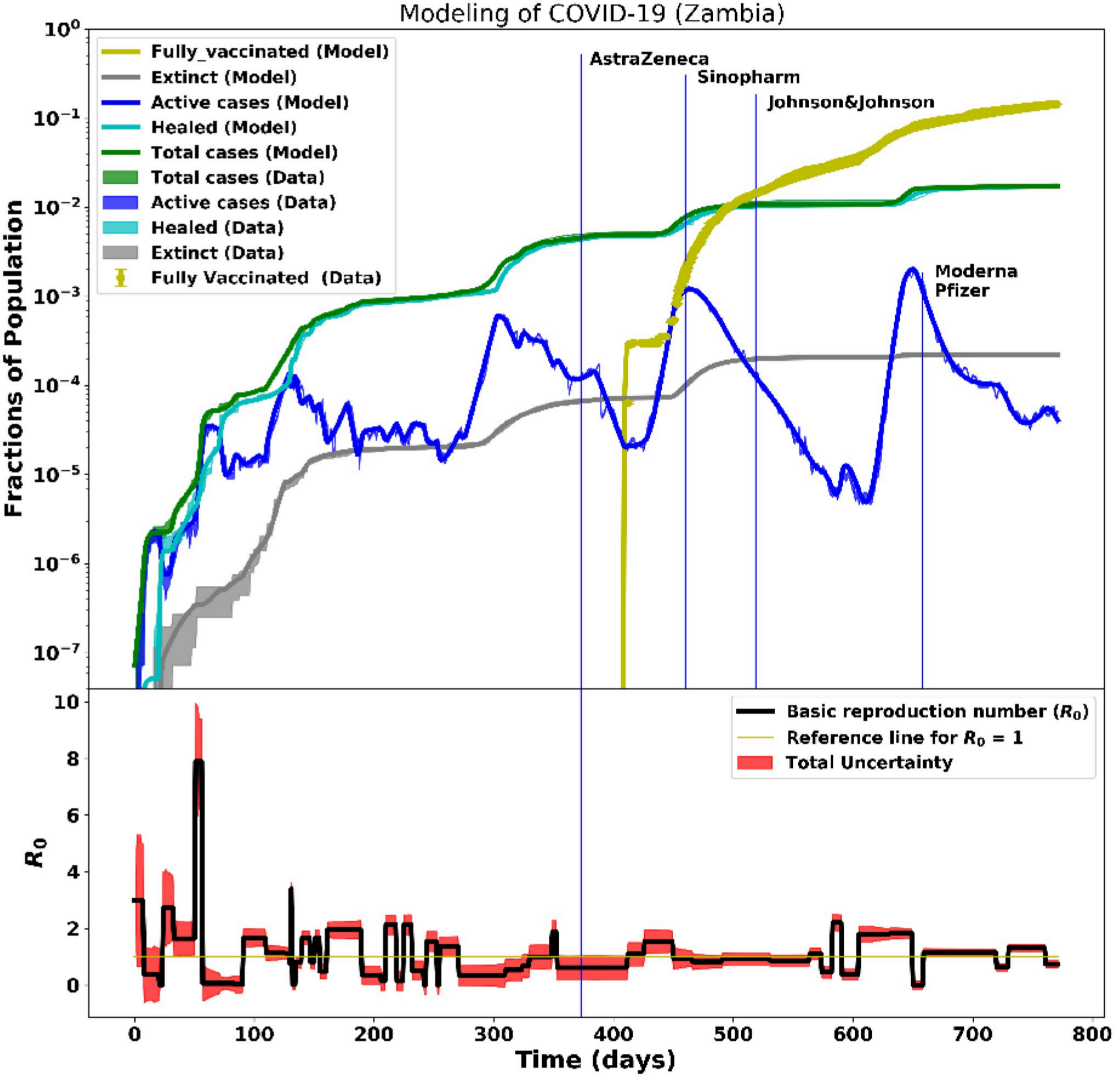
Modelling of ∼2 years of COVID-19 data for Zambia. Day 0 corresponds to the onset of the pandemic on March 18, 2020. The top plots show the data and the fitted model for the active, recovered, death, total cases, and fully-vaccinated cases. Vaccination drives started on April 14, 2021. The bottom plots show the time-dependent basic reproduction number, *R*_0_, and how it varied with time as the pandemic evolved starting at *R*_0_ ∼ 3 at the pandemic onset.

The Zambian data of the first year of the pandemic—before vaccination started—were analysed and presented in Paper I. The government of Zambia, through the Ministry of Health (MoH), officially launched the COVID-19 vaccination campaign on April 14, 2021, at the University Teaching Hospital (UTH) in Lusaka [44]. Zambia received more than 10 million doses of vaccines (Pfizer, Moderna, Johnson & Johnson, Sinopharm, and AstraZeneca) through the COVID-19 Vaccine Global Access (COVAX) program [44–46]. The vaccines were distributed to various vaccination centers across the country through efficient logistics and supply chain management systems. The campaign for the administration of second doses of AstraZeneca, Sinopharm, and Johnson & Johnson was initiated in July 2021. By April 30, 2022, more than a million doses of COVID-19 vaccines were administered to eligible persons (above 18 years), and priority was given to high-risk groups such as the elderly (above 65 years) and people with underlying health conditions [47]. However, Zambia, like other developing countries, experienced significant vaccine hesitancy. To overcome this challenge and achieve 70% herd immunity, the government, through MoH, introduced a door-to-door vaccination campaign and community sensitization on the benefits of vaccination [47]. In addition, non-pharmacological interventions such as keeping a 1-meter social distance, wearing face masks, and hand sanitizing were mandatory in public places. By the time of writing, the 70% vaccination target had not been achieved. Fig 11 (top plot) shows the progression of COVID-19 during the two years of the pandemic. The vaccination drive began on April 14, 2021, and by May 25, 2021, a total of 5,286 were fully vaccinated with Sinopharm and AstraZeneca. In addition, by January 2, 2022, eligible individuals started to receive the booster vaccines, corresponding to a cumulative total of 1,649. However, in about 8 to 9 months following the start of vaccination, *R*_0_ increased twice to approximately 2 due to vaccine hesitancy, lack of strict adherence to COVID-19 protocols such as wearing of masks, hand sanitizing and safe social distancing, as shown in Fig 11 (bottom plot). Finally, around day 700, *R*_0_ reduced significantly to ≈1 following a reduction in active cases and increased recovery. This significant reduction could be attributed to effective measures introduced by the government to curb the spread of COVID-19 such as the mandatory wearing of masks in public places, maintenance of safe social distancing, lockdown measures, and closure of schools and universities, combined with vaccination.

#### 3.1.7 COVID-19 vaccination analysis for South Africa

Four types of COVID-19 vaccines were approved by the South African Health Products Regulatory Authority (SAHPRA), namely, Johnson & Johnson, Pfizer, Sinovac, and AstraZeneca [18]. In this study, only Johnson & Johnsons and Pfizer vaccines were considered due to data availability [48]. Fig 6 (left panel) shows the modelling of the South African data. The first year (up to day ∼365) of the pandemic was studied and extensively discussed in Paper I. The second year of the South African COVID-19 data, with vaccination rollouts, is further discussed in Section 4 as a representation of all other African countries.

In Paper I, we covered the South African COVID-19 data analysis up to the adjusted alert level 3 that was in effect from December 29, 2020—February 28, 2021. Based on the changes in COVID-19 new cases, the government introduced adjusted alert levels defined as follows: Level 1: March 1–May 30, 2021; Level 2: May 31–June 15, 2021; Level 3: June 16–June 27, 2021; Level 4: June 28–July 25, 2021; Level 3: July 26–September 12, 2021; Level 2: September 13–30, 2021; and Level 1: October 1, 2021–April 14, 2022 [18, 49].

On May 3 2022, South Africa confirmed 3, 661, 635 recovered individuals, 100, 377 death cases, ∼17.7 million vaccinated individuals, and 3, 802, 198 active cases [18]. The National State of Disaster was lifted on April 5, 2022 [49]. As of June 9, 2022, 535, 714 COVID-19 hospital admissions recorded [50].

The government had targeted 40 million citizens for vaccination [18]. Healthcare workers were the first group to be vaccinated beginning on February 18, 2021 (day 350) until May 17, 2021 (day 439) under phase 1 of the Sisonke Protocol, which enabled the government to make the Johnson & Johnson vaccine quickly accessible to the public [51, 52]. Whilst the active cases fluctuated during phase 1, the number of deaths, healed, and total cases remained constant, as shown in Fig 6. During Phase 2, which began on May 18, 2021, everyone aged 16 years and above was vaccinated with the first dose of either Johnson & Johnson or Pfizer.

## 4 Discussion

In this study, we have presented an analysis of the COVID-19 data from 7 African countries for two years. Even though the data fitting to the first year of the pandemic (without vaccination and presented in our Paper I) is included, our analysis is mainly focused on the second year of the pandemic with COVID-19 vaccination. To analyze the impact of COVID-19 vaccination, we highlight the case of South Africa where the available data was statistically significant since this data makes up ∼50% of the entire African COVID-19 data. Moreover, it is much larger than all the other countries studied, as described in Section 3.1.7.

**Fig 12:**
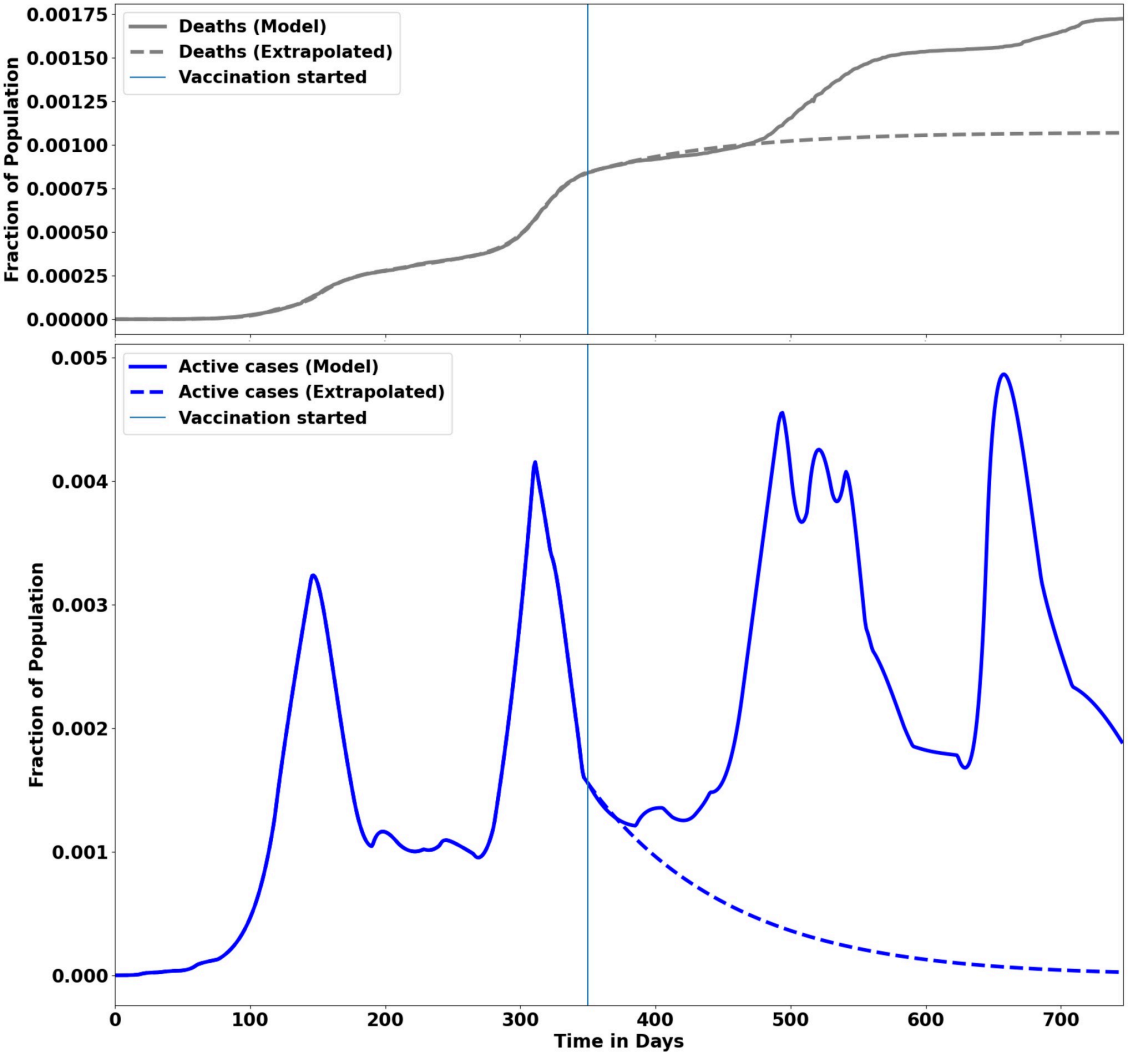
Fitting of the COVID-19 vaccination data for South Africa. Fitting of the model to the death data (top plot) and active data (bottom plot) with extrapolation into the period of the vaccination campaign in South Africa for a period of 2 years, March 2020—March 2022. The vertical dotted line indicates the start of the COVID-19 vaccination campaign around day 350 of the pandemic. Assuming that on day 350, the virus mutations stopped and the social measures were still in place and fully adhered to, then the dynamics of the active cases would follow the dotted blue line, which shows that the number of active cases would decrease with vaccination. Similarly, the number of deaths would be expected to decrease with vaccination as highlighted by the dotted grey line. However, due to new variants and changes in social and COVID-19 measures, the number of active and death cases continued to increase even with vaccination in place, as shown by the solid line.

The vaccination campaign began on Day 349 (February 18, 2021) of the pandemic. Since the number of active cases was declining and *R*_0_ ≈ 0.99, the government relaxed the control measures to alert level 1 on March 1, 2021. The SIDARTHE-V model extrapolation into the period of vaccination, as shown in Fig 12, suggested that the active cases should dwindle and the death rate should plateau over time. However, the relaxation of the control measures by the government without enough vaccinated individuals to reach herd immunity led to the third and fourth waves, as shown in Fig 12, even with vaccination drives in full gear.

The number of people *n* to vaccinate to reach herd immunity:

$$\begin{array}{c} n=N\times (1-\frac{1}{R_{0}})), \end{array}$$ (3)

where *N* is the total population. At the onset of the third and fourth waves, *R*_0_ was estimated at ∼1.4 and ∼2.0, respectively. See Fig 5 (top plot). Assuming *N* = 60 million for South Africa, the number of people to be vaccinated at the beginning of the third and fourth waves was *n*_1_ = 17.1 million and *n*_2_ = 30.0 million, respectively. However, the corresponding number of full-vaccinated people at each wave was 318,670 and 14,031,159, respectively. Although the vaccination was continued, herd immunity was not reached as shown in Fig 6 (left plot). The lack of herd immunity may have contributed to the fifth wave as observed around Day 632, as shown in Fig 5. The effect of vaccination was observed around the fifth wave as shown in Fig 5 whereby:

- the fifth wave was relatively smaller than the previous waves;
- the cumulative deaths were plateauing;
- the daily death counts had fallen;
- and the relaxation of control measures to level 1 without a resurgence of any significant wave.

The impact of vaccination is consistent with what we would expect from a vaccination program and was expected to reduce COVID-19 hospitalizations and deaths. Failure to implement vaccination programs on time can significantly contribute to a rise in the number of infections, increasing the number of hospitalizations and deaths. The basic reproduction number, *R*_0_, which encompasses several pandemic parameters as shown in the SIRDATHE-V model (see Eq 2 and Fig 1) can provide an understanding of the pandemic evolution with control measures and vaccination in place. For example, by comparing the death rates before and after vaccination, the death rate (parameter *τ* in the model) is reduced after and during the vaccination drive, as shown in Fig 5 from ∼ Day 600. This implied that we could have high infection rates (parameters *μ* for undetected asymptomatic and *ν* for detected symptomatic in the model) without people dying in large numbers, as seen from ∼ Day 750. Likewise, a reduction in the infection rates (parameters *μ* and *ν*) would reduce the severity of infections (see the fifth wave in Fig 5).

Even with the relatively low COVID-19 death rates in Africa, the relaxation of the non-pharmaceutical measures by the government at the beginning of the vaccination drive saw a spike in the number of deaths. This could have been drastically reduced had the non-pharmaceutical interventions remained in place even with the commencement of the vaccination program. From the data and SIRDATHE-V model fitting, we conclude that vaccination is an important tool in the fight against COVID-19, and early implementation of the vaccination program could have saved more lives.

## 5 Conclusion

We studied the impact of COVID-19 vaccination in seven African countries; Nigeria, South Africa, Kenya, Ghana, Togo, Mozambique, and Zambia. The SIDARTHE-V model was used in simultaneous fits to the recorded active, recovered, death, and vaccinated data. We observed that combining vaccination drives with control measures to contain the pandemic was essential until herd immunity was achieved. To understand the impact of the vaccination program in Africa, we studied the case of South Africa in more detail since it was the most impacted country in the continent, with statistically significant vaccination data.

Any significant impact of vaccination on the pandemic was observed after almost one year of the drive when ∼1/3 of the population had been fully vaccinated. This was reflected in the significantly reduced daily death counts, the plateauing of the cumulative death rate, and the relaxation of non-pharmaceutical measures without the resurgence of COVID-19 waves. We conclude that vaccination programs must be combined with non-pharmaceutical interventions such as social distancing and masks until enough of the population has been vaccinated such that the relaxation of these measures no longer leads to surges in COVID-19 infections and other similar pandemics.

For the six other countries included in this study, the impact of vaccination was not easy to gauge because of the relatively smaller numbers of both COVID-19 cases and the number of fully vaccinated individuals. However, the conclusion reached in the South African case may apply to other African countries. We recommend that African governments engage with scientists and other researchers early on to safeguard against future pandemics by being prepared to implement social measures and rapid development and deployment of vaccines.
